# Mixed-Mode Bacterial Transmission *via* Eggshells in an Oviparous Reptile Without Parental Care

**DOI:** 10.3389/fmicb.2022.911416

**Published:** 2022-06-28

**Authors:** Teng Li, Yang Yang, Huijun Li, Chunkai Li

**Affiliations:** ^1^College of Resources and Environmental Sciences, Nanjing Agricultural University, Nanjing, China; ^2^Department of Obstetrics and Gynecology, Nanjing Drum Tower Hospital, The Affiliated Hospital of Nanjing University Medical School, Nanjing, China

**Keywords:** maternal cloaca, eggshell, microbiota, symbiont transmission, oviparity, parental care, horizontal and vertical transmission

## Abstract

Symbiotic microorganisms play important roles in maintaining health and facilitating the adaptation of the host. We know little about the origin and transgenerational transmission of symbiotic bacteria, especially in egg-laying species without parental care. Here, we investigated the transmission of bacterial symbionts in the Chinese three-keeled pond turtle (*Mauremys reevesii*), a species with no post-oviposition parental care, by evaluating contributions from potential maternal and environmental sources to eggshell bacterial communities. Using 16S rRNA amplicon sequencing, we established that the bacterial communities of eggshells were similar to those of the maternal cloaca, maternal skin, and nest soil, but distinct from those of surface soil around nest and pond water. Phylogenetic structure analysis and source-tracking models revealed the deterministic assembly process of eggshell microbiota and high contributions of the maternal cloaca, maternal skin, and nest soil microbiota to eggshell bacterial communities. Moreover, maternal cloaca showed divergent contribution to eggshell microbiota compared with two other main sources in phylogenesis and taxonomic composition. The results demonstrate a mixture of horizontal and vertical transmission of symbiotic bacteria across generations in an oviparous turtle without parental care and provide insight into the significance of the eggshell microbiome in embryo development.

## Introduction

Symbiotic relationships between hosts and bacteria are widespread in all animals. Microbial symbionts have significant effects on host health, fitness, and behavior by influencing a range of metabolic, developmental, and physiological processes (Cani et al., 2019; Simon et al., 2019; Kimura et al., 2020; Wang et al., 2020; Hosang et al., 2022). Increasing research has focused on the role of the microbiome in host evolution and its evolutionary consequences, aiming to predict how microbiome variation affects host fitness (Hird, 2017; Yates et al., 2021). Understanding the role of microbial symbionts in animal adaptation and evolution requires knowledge of the origin, maintenance, and transmission of bacteria across generations of animal hosts (Funkhouser and Bordenstein, 2013; Moeller and Sanders, 2020). Although the number of studies of animal-microbe interactions is growing (Montoya-Ciriaco et al., 2020; Perlmutter and Bordenstein, 2020; Xu et al., 2020), more studies in different animal lineages are needed to elucidate the general roles of microbial symbionts in animal adaptation and evolution.

The microbiome during the early stages of life has far-reaching influences on development and fitness of the host later in life. For example, early life disruption of microbiota in tadpoles decreases resistance to parasites in adult frogs (Knutie et al., 2017), and gut bacterial activity contributes to the normal development of ant cuticle (Duplais et al., 2021). Thus, it is of interest to determine how symbiotic associations are established and transmitted across generations. Transmission of symbionts from one generation to the next varies among species and typically follows one of three routes (Bright and Bulgheresi, 2010): horizontal transmission from the environment, as in squids (Nyholm and McFall-Ngai, 2004); vertical transmission from parents, as in insects (Baumann, 2005); and mixed horizontal and vertical transmission, as in humans (Funkhouser and Bordenstein, 2013) and sponges (Reveillaud et al., 2014). Vertical transmission was initially thought to occur only through germ cells, but we now know it includes external maternal transmission, such as through the mouth and skin (Funkhouser and Bordenstein, 2013). Research on the assembly and transmission of microbial symbionts in animals is woefully inadequate, especially in non-human vertebrates.

Transmission of microbial symbionts may also vary among organisms with different reproductive patterns (such as oviparity and viviparity). In viviparous vertebrates, the embryo develops in the mother’s body (usually *in utero*), and the fetus acquires symbiotic microbes through vertical transmission from the mother during development or through horizontal transmission from environmental sources after birth (Funkhouser and Bordenstein, 2013; Moeller et al., 2018). However, for oviparous vertebrates, embryos develop *ex vivo* in the exterior environment. Eggs of oviparous vertebrates serve as essential sites of embryogenesis and function as the dominant carriers for microbial symbionts that are transferred from mother to offspring (Stoddard et al., 2017; Bunker et al., 2021). A healthy fertilized egg is thought to be internally sterile because egg infection can seriously affect embryonic hatchability and the fitness of offspring (Sarmiento-Ramirez et al., 2014a; Hansen et al., 2015; Jacob et al., 2015). Although recent studies of birds’ and squamates’ eggs demonstrated the presence of bacteria *in vivo*, which implies that microbial symbionts may be transmitted from the maternal gut or ovary during egg development (Singh et al., 2014; Trevelline et al., 2018), the eggshell provides a barrier to prevent the passage of microbes. The establishment of beneficial microbes on the eggshell is crucial to embryonic development and successful hatching (Sarmiento-Ramirez et al., 2014b; Peralta-Sanchez et al., 2018; Bunker et al., 2021). For example, establishing some bacteria (e.g., *Enterobacteriaceae*) on the eggshells of lizard (*Sceloporus virgatus*) can improve egg hatching success by protecting the embryos from fungal infection (Bunker et al., 2021). In these respects, we speculate that the eggshell acts as a transgenerational carrier for symbiotic microbes (Dearborn et al., 2017; van Veelen et al., 2018). In oviparous birds, symbiotic microbes are transmitted from mother to eggshell *via* brood patches, nest material, and maternal skin, but not the maternal cloaca (van Veelen et al., 2018). Birds also can transmit microbes to their offspring *via* parental care behaviors (e.g., feeding) (Chen et al., 2020; Li et al., 2022). Transmission of symbiotic microbes across generations has received less attention in oviparous vertebrates without parental care.

Reptiles comprise an ancient vertebrate group in evolutionary history, with a large proportion of oviparous species that provide no parental care (Uetz et al., 2016). Most oviparous reptiles deposit their eggs into a nest that they build using their legs, where the embryos develop until they hatch. They are therefore excellent models to study the transgenerational transmission routes of symbiotic microbes in oviparous vertebrates without parental care. Although some studies have addressed the transgenerational transmission of microbial symbionts in reptiles, most of them focused on viviparous species or the anti-infective function of bacterial communities of eggshells during embryonic development in oviparous species, and few directly examined the intergenerational transmission pathways of microbes (Sarmiento-Ramirez et al., 2014b; Kohl et al., 2017; Bunker et al., 2021). Therefore, do oviparous reptiles without parental care use the eggshell as a transgenerational carrier for symbiotic microbes? What is the route of intergenerational transmission of microbial symbionts? Considering the reproduction mode of oviparous reptiles and the transgenerational transmission routes of symbiotic microbes in animals, we hypothesize that mothers vertically transmit some microbes onto the surface of their eggs from their cloacas during oviposition and that microbes are also horizontally transmitted from nest materials (soil) onto eggshells.

To examine these hypotheses, we applied high-throughput sequencing to 16S rRNA gene amplicons from reptile eggshells and potential sources of bacteria to identify which sources contribute to eggshell bacterial communities in the Chinese three-keeled pond turtle (*Mauremys reevesii*). This turtle is an aquatic emydidae that was once widespread in central and southern China and southeastern Asia (Zhao and Adler, 1993). Due to the threat of habitat destruction and overexploitation, this turtle is difficult to find in the wild but is cultured in commercial turtle farms. Females lay eggs at night during the reproductive season (May to August), taking an average of 1 h to complete an oviposition. The mother digs a cylindrical nest with her hind legs before oviposition and buries the eggs in the nest after oviposition. The average size of the nest is about 12 cm in depth and 8 cm in diameter, and the mean clutch size of this turtle is about nine. After oviposition, the turtle leaves and provides no further care; the embryos develop within the nest until they hatch. We compared α and β diversity of bacterial communities of eggshells and potential sources, including maternal cloaca, maternal skin, nest soil, surface soil around nest and pond water, and then we assessed the phylogenetic structure of eggshell communities to determine whether the process of bacterial assembly on the eggshell exhibited phylogenetic clustering or phylogenetic overdispersion. Whereafter, we applied a SourceTracker model to predict the contribution of each potential source to eggshell bacterial communities. Finally, by comparing the phylogenetic and taxonomic composition of the abundant taxa specifically from each main source, we explored the differences in the contribution of the main sources to eggshell microbiota.

## Materials and Methods

### Sample Collection

**FIGURE 1:**
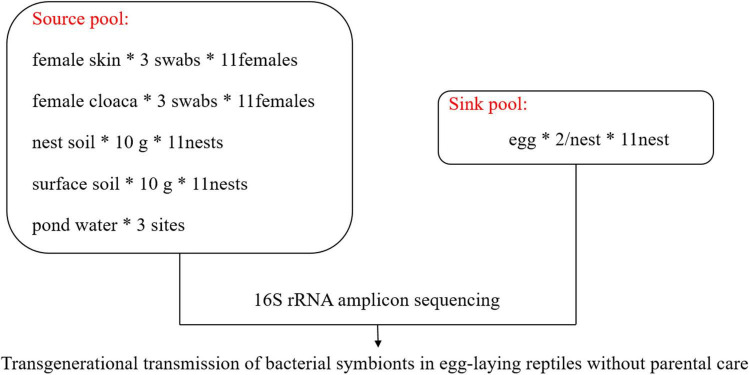
Graphical scheme illustrating experimental design and sample collection. *Multiplication sign.

In July 2020, we monitored the oviposition behavior of females in outdoor ponds (approximately 8 m × 16 m) in Jiaxing (30°15′N, 120°20′E), Zhejiang Province. After females completed oviposition, we captured them as soon as possible. We collected samples from the skin of the back thigh and the cloaca of each female using three sterile PBS moistened swabs, respectively. For skin, the swab was rotated 5–6 times over a ∼2.5 cm^2^ area with gentle pressure. For cloaca, each swab was rotated five times in a slow clockwise motion around the cloaca, so that the swab was coated with cloacal material. Following sample collection, the swab was inserted into the supplied sterile container. The nests were gently excavated and two eggs were randomly collected from each nest with sterile plastic tubes. We collected 10 g of the soil inside the nest and surface soil within a 30-cm radius from the nest center after stirring the soil with a sterile spoon. To avoid interactions between nests that are too close to each other, we only selected those nests that were more than 50 cm away from others. Water was collected at three sites (about 600 ml each site) around spawning ground in the pond using sterile tubes, and ultrafiltration was performed with a 0.22-μm filter to collect the microorganisms in the water. We handled turtles and samples with new, sterile latex gloves that were replaced between samples. All samples were collected within 8 h after oviposition. A graphical scheme illustrating the experimental design and sample collection is shown in Figure 1. In total, we collected samples from 11 female turtles, their 11 nests, and 3 samples of pond water. All samples were placed in liquid nitrogen, transported to the laboratory on dry ice, and stored at –80°C until they were processed for DNA extraction.

### DNA Extraction and Amplification

Shells of the turtle eggs were separated at a clean bench. Shells from two eggs from the same nest were mixed together and then powdered using liquid nitrogen and a sterilized mortar and pestle (Grizard et al., 2014; van Veelen et al., 2018). We removed any cotton fibers left behind by the swabs aseptically. Microbial DNA was extracted using HiPure Soil DNA Kits (Magen, Guangzhou, China) according to the manufacturer’s protocols with minor modifications: the vortex time was set to 8 min and repeated two times to allow complete cell lysis. Meanwhile, we set up a negative control, that is, other conditions and reagents were the same, but no sample was added. After DNA quality checked with NanoDrop 2000 (Thermo Fisher Scientific, United States) and agarose gel electrophoresis, the V3/V4 region of the 16s rRNA gene was amplified using primers 341F (CCTACGGGNGGCWGCAG) and 806R (GGACTACHVGGGTATCTAAT) (Tang et al., 2020). The pooled DNA was amplified with a 50-μL PCR reaction system [in 5 μL 10 × KOD Buffer, 5 μL 2.5 mM dNTPs, 1.5 μL each primer (5 μM), 1 μL KOD Polymerase, and 100 ng template DNA] under the following running conditions: initial denaturation for 2 min at 95°C; 27 cycles of 10 s at 98°C, 30 s at 62°C, and 30 s at 68°C; and a final elongation step for 10 min at 68°C. The amplicons were purified using the AxyPrep DNA Gel Extraction Kit (Axygen Biosciences, Union City, CA, United States) according to the manufacturer’s protocol and quantified using an ABI StepOnePlus Real-Time PCR System (Life Technologies, Foster City, CA, United States). Purified amplicons were pooled in equimolar proportions and paired-end sequenced (2 × 250) on an Illumina platform according to standard protocols.

### Sequence Data Processing

We processed the raw Illumina amplicon reads using QIIME2 Core 2020.8 distribution (Bolyen et al., 2019). After merging paired-end clean reads, we truncated the adapters and filtered the reads with quality (*Q*-value) >20. The clean reads were dereplicated and clustered by open-reference OTU-picking (Operational Taxonomic Unit) [using Greengenes reference set (v. 13.8) (McDonald et al., 2012)] using 97% identity by q2-v search. Chimeras were detected and removed, and representative sequences of OTUs were taxonomically annotated using a pre-trained Naive Bayes classifier (Werner et al., 2012) based on the bacterial 16S rRNA Greengenes 13_8 99% OTUs. We removed singletons and OTUs belonging to Archaea, chloroplasts, and mitochondria. A phylogenetic tree was generated from the filtered alignment using QIIME2. Finally, our 16s rRNA sequencing produced 39,552 OTUs; sequence coverage ranged from 35,480 to 103,661 reads per sample.

### Statistical Analysis

α-Diversity indices (ACE, Shannon index) and β-diversity metrics (Bray-Curtis dissimilarity and weighted UniFrac distance) were calculated using *phyloseq* [v. 1.32.0 (McMurdie and Holmes, 2013)], *picante* [1.8.2 (Kembel et al., 2010)], and *vegan* (v. 2.5-6), with the OTU table rarefied to 10,000 reads per sample because the Shannon diversity leveled off at 10,000 reads (Supplementary Figure 1). According to the normality of distributions and homogeneity of the data, non-parametric test (Kruskal–Wallis test) was applied to explore the differences in the alpha-diversity of bacteria between different groups. Pairwise Wilcox tests were conducted to test the differences of bacterial α-diversity between eggshells and each potential source (with bonferroni-adjusted *P*-values). The non-metric multidimensional scaling (NMDS) ordination approach based on Bray-Curtis dissimilarities and weighted UniFrac from the OTU-level table was conducted using the R package *phyloseq*. We used a permutational multivariate analysis of variance (PERMANOVA) to test the significance of differences in bacterial communities between different groups. The pairwise differences of the bacterial communities were detected using the R package *RVAideMemoire* (v. 0.9-79).

We explored the relative abundances of bacterial phyla between eggshells and each potential source. To further measure the specificity and fidelity of the most prevalent bacterial taxa (at the family level, average abundance >0.1%) in eggshells from other sample types, we determined its indicator value index (IndVal) with the R package *labdsv* (2.0-1). IndVal takes into account the relative abundance of the taxon in a certain community and its frequency of occurrence in all communities, with larger values indicating greater specificity of that taxon in a given sample type. To determine the assembly process of the bacterial community on eggshells, mean nearest taxon distance (MNTD) and nearest taxon index (NTI) were computed with 999 randomizations using the R package *picante* (1.8.2) (Kembel et al., 2010; Stegen et al., 2012; Dini-Andreote et al., 2015). NTI > 2 or mean NTI > 0 indicates that coexisting taxa are more closely related than might be expected by chance (phylogenetic clustering). NTI < −2 or mean NTI < 0 suggests that coexisting taxa are more distantly related than might be expected by chance (phylogenetic overdispersion) (Kembel, 2009; Stegen et al., 2012). After that, SourceTracker, a Bayesian community-level microbial source-tracking tool (Knights et al., 2011), was employed to explore the proportion of eggshell microbiota (sink pool) that originated from potential sources (cloaca, skin, nest soil, surface soil, and pond water) with default model parameters. We used a Kruskal–Wallis test to estimate the differential contribution of potential sources to the eggshell bacterial community and a Dunn’s test for multiple group comparisons. Finally, we analyzed the differences of phylogenetic relationship and comosition of the dominant bacteria in eggshell communities that specifically originate from each main source (that is, the OTU is found only in eggshell and a given sample group), removing low-abundance OTUs (average abundance <0.01%) in eggshells. Using the identified sequences, a circular phylogenetic tree was constructed with MEGA X using the maximum likelihood method and displayed using iTOL (Kumar et al., 2018; Letunic and Bork, 2021).

## Results

### Eggshell Bacterial Communities More Closely Resemble Those of Maternal Sources

**FIGURE 2:**
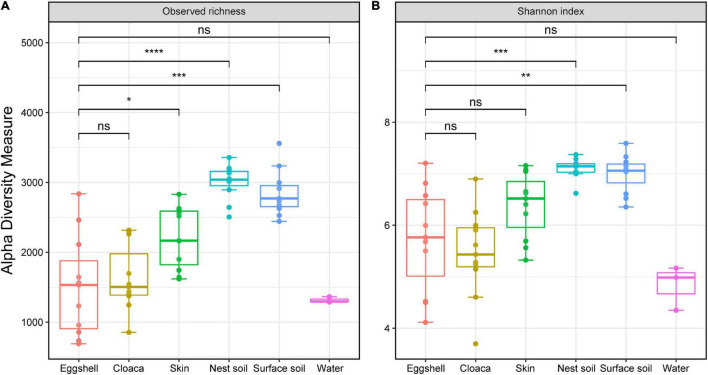
α-Diversity across eggshells and potential sources of microbes in Chinese three-keeled pond turtle. Observed richness **(A)** and Shannon diversity **(B)** of microbiomes from eggshells, maternal sources (cloaca and skin), and environmental sources (nest soil, surface soil, and pond water). Significance of comparisons between eggshells and other communities is shown (Wilcox test, FDR-corrected *P*-values, *P < 0.05, **P < 0.01, ***P < 0.001, ns not significant). Box centerlines, edges, whiskers, and points signify the median, interquartile range (IQR), 1.5 × IQR, and >1.5 × IQR, respectively. ****P < 0.0001.

We found the bacterial community varied across different sample types. The OTU richness of eggshells did not differ from that of the cloaca and pond water but was lower than that of maternal skin, nest soil, and surface soil (Figure 2A). Similarly, the bacterial community of nest soil and surface soil had a higher Shannon diversity than that of eggshell. The diversity of eggshell bacterial communities was not significantly different from that of the cloaca, maternal skin, and pond water bacterial communities (Figure 2B).

### Eggshell Bacterial Compositions Are More Similar to That of Maternal Sources and Nest Soil

**FIGURE 3:**
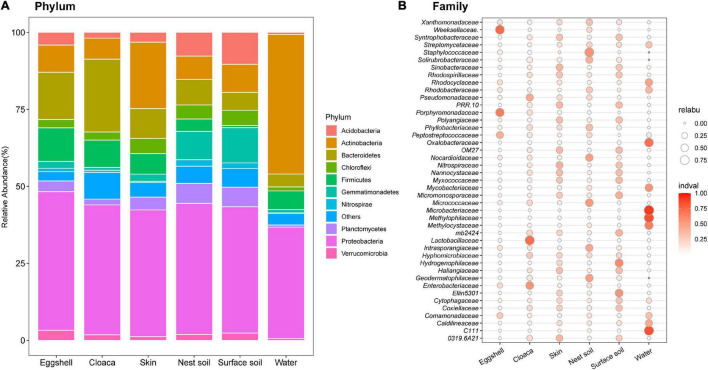
Compositions of bacterial communities from eggshells and potential sources. **(A)** The 10 most abundant phyla among eggshells and maternal and environmental sources. Different colored portions of the bars represent the relative abundance of a given phylum for each group. **(B)** Indicator analysis across all sample types at the family level, after removing species with average abundance <0.1% in eggshells. The size of the bubble represents the relative abundance, and the color intensity indicates the specificity of the species in the sample type.

Of the 67 total identified bacterial phyla, 10 dominated the eggshell community (average accumulative abundance = 94.5%). Relative abundances of these 10 phyla were variable in maternal and environmental communities (Figure 3). *Proteobacteria* was the most prevalent phylum in communities from eggshells and all potential sources. *Bacteroidetes*, *Firmicutes*, and *Actinobacteria* had greater abundance in eggshell and maternal sources than in environmental sources, except for the dominant *Actinobacteria* in pond water (Figure 3A). We determined the indicator value (IndVal) to represent the specificity of a taxon (at the family level) to a given sample type (FDR-corrected *P* < 0.05). The indicator taxa varied in different sample types (Figure 3B). Most of the taxa that were specific to eggshell were also the indicator taxa in maternal and nest soil communities, including *Weeksellaceae* (*Proteobacteria*), *Porphyromonadaceae* (*Bacteroidetes*), *Peptostreptococcaceae* (*Firmicutes*), and *Enterobacteriaceae* (*Proteobacteria*). Some taxa were prevalent in surface soils but not in eggshell, such as *mb2424* (*Acidobacteria*), *Hydrogenophilaceae* (*Proteobacteria*), and *Ellin5301* (*Gemmatimonadetes*). In addition, the most abundant taxa that were specific to pond water did not thrive on eggshells. These results suggest that the bacterial taxa thriving on eggshells shortly after oviposition originated from a mixture of maternal and environmental sources, except for pond water.

### Bacterial Community Structure in Eggshells Is Phylogenetically More Similar to Maternal Sources

**FIGURE 4:**
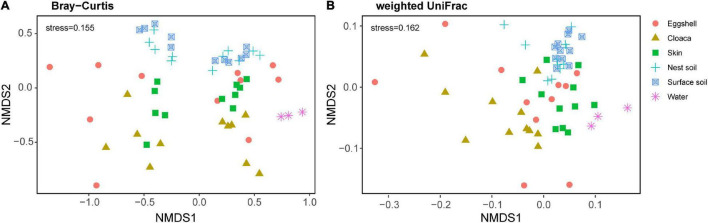
β-Diversity of the bacterial community across eggshells and maternal and environmental samples. Non-metric multidimensional scaling (NMDS) plots based on Bray-Curtis distances **(A)** and weighted UniFrac distances **(B)** of eggshell and source communities. Each point represents the bacterial community of a sample; the color and shape of the point represent different sample types.

We examined the structural similarities of bacterial communities between eggshells and maternal and environmental sources by comparing taxonomic and phylogenetic composition through non-metric multidimensional scaling (NMDS) analysis. Bray-Curtis dissimilarities and weighted UniFrac distances strongly varied according to sample type (Bray-Curtis, *F*_5, 52_ = 3.01, *P* = 0.001; weighted UniFrac, *F*_5, 52_ = 5.41, *P* = 0.001). Pairwise comparisons showed that the structure of the eggshell bacterial community was separate from the structures of bacterial communities from potential sources (Supplementary Table 1). Even so, the bacterial community structure of eggshell was more similar to those of maternal cloaca and maternal skin than to those of environmental sources (Figure 4).

### Phylogenetically Clustered Eggshell Bacterial Communities

**FIGURE 5:**
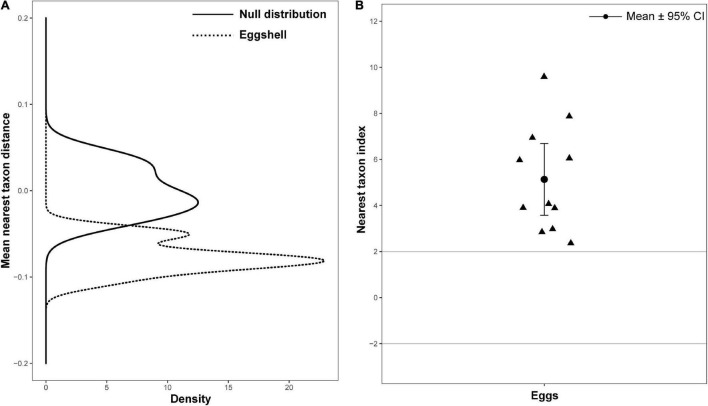
Bacterial community phylogenetic structure of eggshell. **(A)** Mean nearest taxon distance (MNTD) distributions of phylogenetic structure of eggshell bacterial community (dotted line) deviation from null distribution (solid line, with 999 randomizations). **(B)** Nearest taxon index (NTI) for eggs of Chinese three-keeled pond turtle. Each triangle represents one sample; data are expressed as mean ± 95% CI.

Null model approaches, based on phylogenetic community structure, were used to determine whether the assembly process of the eggshell bacterial community reflected phylogenetic clustering (e.g., selective niche) or phylogenetic overdispersion (e.g., random dispersal). The distributions of MNTD deviated significantly from the permutated null distribution in eggshell (Figure 5A). The NTIs of all eggshell communities were greater than 2, with an average value of 5.56, significantly more than 0 (*P* < 0.001) (Figure 5B). These results indicate that the assembly of bacteria on eggshells may be shaped by both environmental and biotic conditions (Stegen et al., 2012).

### Maternal Cloaca, Skin, and Nest Soil Are Three Main Sources of Eggshell Bacterial Communities

**FIGURE 6:**
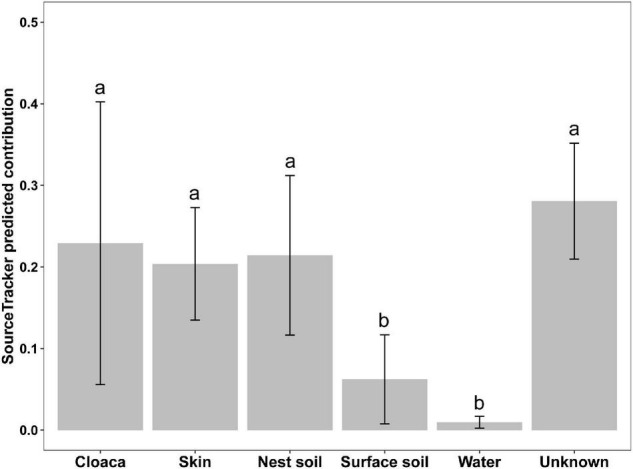
Predicted contributions of maternal and environmental sources to eggshell bacterial communities. Different lowercase letters indicate a statistically significant difference (Dunn’s test, FDR-corrected *P* < 0.05). Data are expressed as mean ± 95% CI.

We used a Bayesian community-level microbial source-tracking tool to investigate how maternal and environmental sources contributed to the bacterial community of eggshell. The model revealed different proportional contributions to eggshell communities from all potential sources (Figure 6, Kruskal–Wallis, χ^2^ = 30.97, df = 5, *P* < 0.001). Further analysis revealed a major role for maternal sources [cloaca (22.91%) and skin (20.38%)] in shaping eggshell bacterial communities, which corroborates the patterns observed in bacterial community structure analysis. Among environmental sources, nest soil (21.43%) was a main contributor to eggshell bacterial communities, but surface soil around the nest (6.22%) and pond water (0.98%) were not. The results of Dunn’s z statistic test are shown in Table 1. In all, the SourceTracker model identified maternal cloaca, skin, and nest soil as three dominant sources of eggshell bacterial communities, despite the significant differences in structure between eggshell bacterial communities and those of potential sources. These results imply that bacteria are transferred between generations of this turtle that lacks parental care after oviposition *via* a mixture of vertical and horizontal transmission (Figure 6 and Table 1).

**TABLE 1:** Pairwise comparison of the contribution ratios of all predicted sources by SourceTracker (P-values are in parentheses).

	Cloacal gut	Skin	Nest soil	Surface soil	Unknown
Skin	−0.52 (0.347)				
Nest soil	−0.37 (0.382)	−0.16 (0.438)			
Surface soil	1.97 (**0.045**)	2.49 (**0.021**)	2.34 (**0.021**)		
Unknown	−1.58 (0.095)	−1.06 (0.181)	−1.21 (0.169)	−3.55 (**0.001**)	
Pond water	3.14 (**0.003**)	3.66 (**<0.001**)	3.51 (**<0.001**)	1.17 (0.165)	4.72 (**<0.001**)

*Statistically significant difference (Dunn’s test, FDR, P < 0.05) are represented in bold.*

### Maternal Cloaca Showed Divergent Contribution to Eggshell Bacterial Communities Than Two Other Main Sources

**FIGURE 7:**
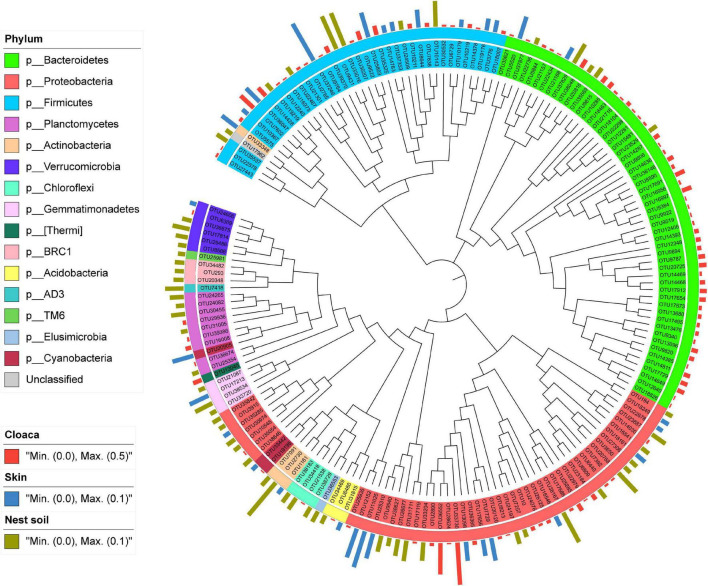
Phylogram and relative abundance of eggshell bacterial communities specifically associated with the three main sources. Branch length is not proportional to the genetic distance. The taxonomic affiliation at phylum level of each specialist OTU is indicated by the color range in the left panel and within the tree. The relative abundance of species in eggshell is indicated in bars outside of the tree, with different colors representing different sources.

After data filtering, we detected 185 OTUs specifically associated with three main sources. Among these OTUs, 98 OTUs (52.97%) were associated with cloaca, 30 OTUs (16.22%) with maternal skin, and 57 OTUs (30.81%) with nest soil. Phylogenetic analysis showed that most of the OTUs belonging to cloaca showed high clade specificity, while the OTUs associated with maternal skin or nest soil were broadly distributed across the phylogenetic tree. Besides, some OTUs belonging to cloaca were also clustered together with maternal skin and nest soil (Figure 7). At the phylum level, the abundant taxa associated with cloaca belong to *Bacteroidetes*, and the dominant taxa related to maternal skin are *Proteobacteria* and *Firmicutes*. *Proteobacteria* comprised the most abundant bacteria that associated with nest soil. Compared with other sources of bacteria, the differences in phylogenetic composition of abundant taxa suggest the specificity of the contribution of maternal cloaca-derived bacteria to eggshell microbiota.

## Discussion

By comparing the diversity, composition, and assembly of bacterial communities of eggshells and potential sources of microbes, and by predicting the contributions of potential sources with a Bayesian approach (SourceTracker model), we demonstrated a mixed horizontal and vertical mode of transmission of symbiotic bacteria across generations in an oviparous vertebrate (*M. reevesii*). Specifically, we demonstrated vertical transmission from maternal cloaca and skin, as well as horizontal transmission from nest soil. These findings are consistent with our hypothesis and reflect disparate routes of microbial transmission among oviparous vertebrates without parental care. The results enrich our understanding of transgenerational transmission of microorganisms in vertebrates.

Our findings support the speculation that eggshells function as carriers of maternal gut symbionts and play a role in transgenerational offspring inoculation, at least in oviparous reptiles without parental care. These results echo previous studies on the important role of eggshell microorganisms in embryonic development and anti-infection and jointly explain the impacts of eggshell and eggshell microbiota on the evolutionary history of animals (Peralta-Sanchez et al., 2018; Bunker et al., 2021). As eggs are directly exposed to microbes in the nest, it is generally believed that the richness and diversity of eggshell microbiota will be higher than those of maternal cloaca microbiota (van Veelen et al., 2018). However, we found similar richness and diversity of bacterial communities of eggshell and maternal cloaca in our study. This similarity may be caused by the captivity and dietary diversity associated with farms, which have profound effects on the diversity of gut microbiota in animals (Zarrinpar et al., 2014; Kohl et al., 2017; Montoya-Ciriaco et al., 2020). For example, captivity increased the community abundance and influenced community structures of gut microbiota in crocodile lizards (*Shinisaurus crocodilurus*) (Tang et al., 2020). The increased richness and changed structural of the cloacal community may make it more similar to the eggshell bacterial community. In this study, we sampled within 8 h after oviposition, when microbiota have just colonized the surface of the egg. We do not know which bacteria can continue to survive and thrive on eggshells or whether these thriving bacteria cross the eggshell barrier, as its permeability increases with the absorption of calcium during embryonic development to help embryos shape their symbiotic microbiota (Sexton et al., 2005; Solomon, 2010; Starck et al., 2021). Illustrating the assembly dynamics of microbiota on eggshells during embryo development until hatching will be essential to better understand their function in transgenerational transmission of symbiotic microbiota.

The eggshell bacterial community of *M. reevesii* was largely dominated by the phyla *Proteobacteria*, *Bacteroidetes*, *Firmicutes*, and *Actinobacteria*, similar to observations in previous studies in lizards and turtles (Sarmiento-Ramirez et al., 2014b; Montoya-Ciriaco et al., 2020; Garcia-Martin et al., 2021). These phyla protect eggs from fungal infection during embryo development in fishes, reptiles, amphibians, and some invertebrates (Liu et al., 2014; Sarmiento-Ramirez et al., 2014b; Walke and Belden, 2016; Florez et al., 2018; Nyholm, 2020). For instance, some genera of *Actinobacteria*, isolated from sea turtle (*Eretmochelys imbricata*) eggshell, inhibit hyphal growth of the pathogen *Fusarium falciforme*, which poses a major threat to sea turtles (Sarmiento-Ramirez et al., 2014b). Vertical transmission of the symbiotic bacterium *Enterobacteriaceae* (belong to *Proteobacteria*) protects embryos from fungal infections in the striped plateau lizard (Bunker et al., 2021). Coincidentally, *Enterobacteriaceae* were also prevalent on *M. reevesii* eggshell and maternal cloaca. These findings suggest that the dominant bacteria on *M. reevesii* eggshell, vertically transmitted from maternal cloaca, may help the embryos resist fungal infections during development, improving the fitness of the offspring (Nyholm, 2020; Bunker et al., 2021).

Our data showed significant phylogenetic clustering in the eggshell bacterial community, which means its structure is shaped by both environmental and biotic conditions, such as eggshell niche-based selection and competition among bacterial taxa, rather than by random processes of dispersal and drift (Dini-Andreote et al., 2015). In birds, the eggshell’s cuticle or the antibacterial components that are adhered by its special structure, which are secreted from uropygial, act as a selective force for microbes to help establish a healthy microbiota on eggshell (Martin-Vivaldi et al., 2014; D’Alba et al., 2016). It is unclear whether there is a similar mechanism in reptiles, especially in those with no parental care. Nevertheless, the similarity in bacterial community composition of eggshells and maternal sources (cloaca and skin) and environmental sources (nest soil), along with the predominantly phylogenetically clustered process of eggshell bacterial communities, reveals a mixture of horizontal and vertical transmission routes of symbiotic bacteria across generations in an oviparous turtle (*M. reevesii*).

Additionally, we quantified the contributions of potential sources to eggshell bacterial communities using the SourceTracker model. Maternal cloaca, maternal skin, and nest soil were the three main sources of bacteria on eggshells, accounting for 64.72% of the community. Furthermore, maternal cloaca-derived bacteria on eggshells showed a special phylogenetic composition of abundant taxa compared with two other main sources. These results were congruent with findings in lizards, squids, and sponges, but distinct from results in birds, showing that eggshells do not carry maternal cloaca bacteria shortly after oviposition (Reveillaud et al., 2014; van Veelen et al., 2018; Kerwin et al., 2019; Bunker et al., 2021). This difference can be attributed to the role of different parental care behaviors in the transgenerational transmission of microbial symbionts in egg-laying species. For example, birds transmit microbes mainly through hatching and breeding behavior, rather than during oviposition (van Veelen et al., 2018; Chen et al., 2020). Incubation behavior in birds can reduce the growth of microorganisms on the eggshell, thereby reducing the opportunity for *trans*-shell infection and increasing hatching success (Cook et al., 2005; Svobodova et al., 2019). By contrast, in egg-laying species with no parental care, colonization of the egg before eggshell formation or colonization of the eggshell when eggs pass through the cloaca are the main ways that mothers transmit microbes to their offspring (Trevelline et al., 2018; Bunker et al., 2021). Establishing a healthy microbiota is important for preventing fungal infection during development, as the eggs are directly exposed to soil microbes, and the warm, moist conditions inside the nest are particularly suitable for the growth of fungi, which are the main cause of egg-hatching failure (Sarmiento-Ramirez et al., 2014a,b; Nyholm, 2020). In our study, approximately 21.43% of bacteria on eggshells were transmitted horizontally from soil within the nest. This finding is similar to a previous study in birds showing that nest material was a main source of eggshell microbes (van Veelen et al., 2018; Chen et al., 2020). We do not know whether these horizontally transmitted microbes function to help embryos develop normally or to help juveniles construct healthy microbiomes. Even so, some studies have shown that horizontally transmitted symbiotic bacteria of beetles could facilitate their defense against antagonists (Florez et al., 2018; Waterworth et al., 2020). In these respects, we speculate that eggshell microbes transmitted from maternal and environmental sources form a healthy microflora through their interactions and the selection of the eggshell niche and further facilitate the development of embryos and the construction of juvenile symbiotic microbiomes. Even so, further investigations are needed to validate this speculation, not only with experimental application of artificially assembled microbes, but also in many more oviparous species across different animal lineages.

## Conclusion

This study supports the hypothesis that eggshells function as potent carriers of maternal gut symbionts and demonstrates mixed horizontal and vertical transmission of symbiotic bacteria across generations in an oviparous turtle without parental care (*M. reevesii*). The results of this study enrich our understanding of the transgenerational transmission of microbial symbionts in species with different reproductive modes and lay a foundation for further exploring the role of microbial symbionts in animal adaptation and evolution. Further research is needed to unveil the general routes of microbial symbiont transmission across more animal lineages. In addition, the dynamics of the eggshell bacterial community and its subsequent impacts on embryo development and performance are not well understood, especially in species that lack parental care.

## Data Availability Statement

The data presented in this study are deposited in the National Center for Biotechnology Information (NCBI) SRA database, accession number PRJNA762989.

## Ethics Statement

The animal study was reviewed and approved by the Animal Care and Use Committee at Nanjing Agricultural University.

## Author Contributions

TL and YY conceived the project. TL and CL collected the samples. TL, HL, and CL analyzed the data. TL, YY, and HL wrote the manuscript. All authors approved the final version of the manuscript.

## Conflict of Interest

The authors declare that the research was conducted in the absence of any commercial or financial relationships that could be construed as a potential conflict of interest.

## Publisher’s Note

All claims expressed in this article are solely those of the authors and do not necessarily represent those of their affiliated organizations, or those of the publisher, the editors and the reviewers. Any product that may be evaluated in this article, or claim that may be made by its manufacturer, is not guaranteed or endorsed by the publisher.
