# Supplementary material for: Mixed-Mode Bacterial Transmission via Eggshells in an Oviparous Reptile Without Parental Care
Source: Front Microbiol. 2022 Jun 28;13:911416. doi: 10.3389/fmicb.2022.911416 (PMC9273969; doi:10.3389/fmicb.2022.911416)
Supplement: Supplementary file 1 [file Data_Sheet_1.PDF]

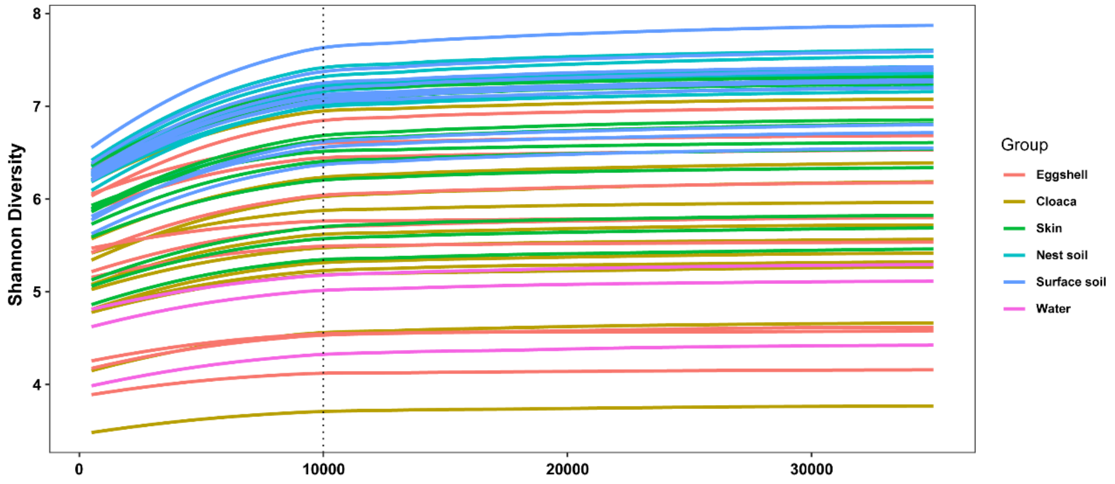

Fig. S1 Rarefaction curves for Shannon diversity of all samples. Samples of eggshells and potential sources are colored by sample type. Shannon diversity reached a plateau in all samples at 10000 reads per sample (dashed line)

Table S1 PERMANOVA analysis of comparisons between eggshell communities and maternal and environmental source communities

Sample type	Bray-Curtis			Weighted UniFrac		
	F	R^2	*P*	*F*	R^2	*P*
Cloacal gut	2.82	0.12	**0.003**	2.72	0.12	**0.014**
Skin	1.77	0.08	**0.006**	2.70	0.12	**0.004**
Nest soil	1.83	0.08	**0.006**	3.25	0.14	**0.004**
Surface soil	2.10	0.09	**0.004**	5.65	0.22	**0.003**
Pond water	2.91	0.20	**0.005**	3.38	0.22	**0.007**

Statistically significant difference ($P < 0.05$) are represented in bold
